# Differences in the amplitude of dynamic low-frequency fluctuations in primary angle-closure glaucoma are associated with gene-molecular multi-omics

**DOI:** 10.3389/fmed.2025.1679910

**Published:** 2025-09-17

**Authors:** Yuan Hu, Rui-Yang Hu, Hai-Jun Yang, Ting-Ting Xu

**Affiliations:** ^1^Nanchang Bright Eye Hospital, Nanchang, China; ^2^School of Ophthalmology and Optometry, Jiangxi Medical College, Nanchang University, Nanchang, China

**Keywords:** dynamic low-frequency fluctuation amplitude, primary angle-closure glaucoma, gene, receptor/transmission, fMRI

## Abstract

**Objective:**

Primary angle-closure glaucoma (PACG), an incurable ophthalmic disease, is a serious risk to human visual health. Previous studies have demonstrated a strong link between PACG and neuroimaging changes in the brain. This study utilizes dynamic low-frequency fluctuation amplitude (dALFF) with the aim of resolving the potential dynamic alterations in neurological function in PACG and integrating transcriptomics profiles with spatial distribution characteristics of neuromodulatory receptors/transporters to systematically elucidate the underlying neurophysiopathological mechanisms.

**Methods:**

We used sliding time windows of 30TR, 50TR and 80TR to calculate dALFF values and performed partial least squares regression (PLS) analysis of t-values after two-sample test of dALFF values under the sliding window of 50 TR against the Allen Human Brain Atlas (AHBA) to screen genes. Enrichment analysis, tissue-specific expression analysis and protein–protein interactions (PPI) network construction were implemented. The t-values were also analyzed for spatial correlation with neurotransmitter receptor/transporter density profiles distributed throughout the brain.

**Results:**

The two-sample tests under three sliding windows revealed extensive brain alterations in PACG and each abnormal brain region showed elevation (the Gaussian Random Field method, with significance at the voxel level set at *p* < 0.005 (two-tailed) and at the cluster level at *p* < 0.01), which was mainly in the occipital lobe and angular gyrus. Enrichment analysis were mainly “regulation of neuron projection development” and “membrane organization” pathways (*p* < 0.05, no corrected). Specific expression analysis revealed that the relevant genes were involved in all stages of thalamic development. PPI analysis demonstrated the role of PACG-associated genes in the formation of functional network. Neurotransmitter receptor/transporter correlation analysis revealed significant associations with 5-HT4R and mGlu5R (*p* < 0.05, FDR corrected).

**Conclusion:**

The present study reveals that a wide range of brain regions in PACG patients show significant functional remodeling, elucidating the molecular regulatory network behind this type of pathological alteration.

## Introduction

Primary angle-closure glaucoma (PACG) is a type of ocular disease characterized by anatomical atresia of the anterior chamber angle. Its pathogenesis stems from mechanical occlusion of the angle triggered by an abnormal iris-cornea anatomical position, which in turn leads to dysfunction of the atrial drainage system and an abnormally high intraocular pressure (IOP), resulting in optic nerve damage as a result of this glaucomatous type of glaucoma. The core anatomical hallmarks of the disease include a markedly shallow anterior chamber (ACD < 2.5 mm), a narrow angle (<20°), and anterior dislocation of the lens, which together result in frequent iris-to-cornea contact (ITC), a set of features that have been confirmed by several imaging studies (1–3). Epidemiological data show a significant gender imbalance and heterogeneity across geographic regions, with East Asian populations having a particularly high prevalence; of particular interest is the fact that the risk of the disease in the female population can be up to one and a half times higher than that in the male population, a gender-preference phenomenon that has been demonstrated in multicenter epidemiological studies (4–6). PACG can cause severe vision loss in patients because of its irreversible damage to the optic nerve. Cause severe vision loss, visual field defects, and even blindness (7–10). Therefore, early detection, diagnosis and treatment are the most important measures to prevent blindness in PACG patients.

In recent years, major recent technological innovations have propelled the progressive development of magnetic resonance imaging (MRI) as a highly clinically valuable tool for the early prediction of disease (11–15). As an important branch of this technique, functional magnetic resonance imaging (fMRI) aims to reveal pathological changes in the topology of functional neural networks in the brain. The technique employs a non-invasive monitoring mode to pinpoint the synchronized activation characteristics of neuronal clusters by capturing real-time hemodynamic parameters and metabolic level fluctuations in brain regions. Relying on the physical basis of blood oxygen level-dependent (BOLD) effect, this technique has successfully achieved millimeter-scale spatial resolution and sub-second temporal resolution, which provides a technical guarantee for capturing transient neural activity characteristics (16, 17). A technological framework based on fMRI has been established in recent years to systematically study the phenomenon of CNS reorganization in patients with PACG (18–20). In terms of brain structure, Jiang et al. found that PACG patients had varying degrees of altered gray matter volume across a wide range of brain regions (21). From a functional brain perspective, both Wang et al. and Zhong et al. used independent component analysis (ICA) to find various network alterations in PACG patients (20, 22). In addition, Wang et al. explored the connectivity of the PACG brain network in depth and found that PACG patients showed lower strength than HC patients, whether it was the functional connectivity (FC) network, the structural connectivity (SC) network, or the FC-SC coupling (23). As a result, patients with PACG have varying degrees of alterations in brain structure and function.

Brain-wide slow oscillations are a characteristic feature of the mammalian neocortex that occurs spontaneously in the virtual absence of sensory stimulation. Amplitude of low-frequency fluctuations (ALFF) is a method of blood oxygen level-dependent signal analysis based on resting-state functional magnetic resonance imaging, which quantitatively characterizes the energy intensity of spontaneous neural activity in brain regions by calculating the mean of the square root of the power spectrum of the BOLD signal in a specific low-frequency frequency band (typically ranging from 0.01 to 0.1 Hz) (24–26). It reflects the energy level of local neuronal activity in the resting state of the brain, and shows a significant positive correlation with the amplitude of spontaneous neural oscillations in the baseline state (27, 28). ALFF has been used to investigate abnormal neurological alterations in a variety of disorders and for early diagnosis of diseases, e.g., Alzheimer’s disease (29, 30), depression (31, 32), and so on. In addition, Huang et al. found the presence of ALFF abnormal values in a wide range of brain regions in PACG patients (33); Li et al. and Jiang et al. found extensive brain region alterations using ALFF in different bands (full, slow 4, and slow 5 bands) for PACG (34, 35). However, existing studies have mainly focused on the static characterization of rs-fMRI, and have not yet systematically revealed the dynamic spatiotemporal properties of neural activity signals during PACG pathology.

The current research on rs-fMRI has gradually formed the consensus that the “resting state” subjects are not completely physiologically quiescent, and that their functional neural networks still maintain significant active representations. Even in the absence of external cognitive tasks, the cortex maintains complex neural information processing mechanisms. For example, the default mode network (DMN) is continuously active at rest and forms a dynamic equilibrium with the central executive network (CEN) and salient network (SN) (36, 37). In particular, rs-fMRI signals are not only biological markers of neural activity, but also the result of confounding factors such as neurovascular coupling mechanisms, physiological artifacts (e.g., cardiac/respiratory rhythms), and head micromotion (38–40). This suggests that the energy metabolism level of neuronal population activity is not a constant parameter, but a dynamic process with significant time-varying characteristics, even under resting conditions. In recent years, academics have paid more and more attention to the research paradigm of dynamic characterization metrics of functional brain networks, among which the dynamic low-frequency amplitude fluctuation (dALFF), as a typical representative of innovative metrics, has gained wide attention in the academic community (41, 42). Unlike the traditional static ALFF analysis strategy that incorporates full-time data into the calculation, dALFF captures the dynamic features of brain activity by segmenting the entire functional magnetic resonance imaging (fMRI) time series into multiple time windows, calculating the ALFF values within each window, and analyzing the variance or variability of these values (43, 44). Today, dALFF has been widely used in a variety of neuropsychiatric disorders (41, 45) such as Parkinson’s disease (46), Alzheimer’s disease (47), and generalized anxiety disorder (35). Given that PACG patients have been shown to be characterized by resting-state ALFF abnormalities in multimodal brain regions, the present study intends to systematically investigate the specific patterns of alterations in the time-varying features of their neurological functional networks by means of an innovative dALFF approach.

Significantly, the pathologic process of PACG exhibits a strong association with specific loci. Genome-wide association studies (GWAS) have revealed that polymorphic profiles at loci such as ABCA1, PMM2, PLEKHA7, and COL11A1 show significant correlations with disease risk (48–50). Accumulating evidence-based medicine evidence confirms a profound spatial correlation between functional connectivity patterns of macroscopic brain networks and region-specific gene expression profiles (51–53). In the field of joint neuroimaging-transcriptome research, the Allen Human Brain Atlas (AHBA) has been widely adopted as a benchmark dataset (54–56). The AHBA dataset maps the region-specific expression profiles of the human brain covering 18,686 genes through transcriptomic analysis of postmortem brain tissue. The establishment of whole-brain gene expression profiles bridges the gap between connectome and transcriptome studies (57–60). There have been many previous studies utilizing AHBA and neuroimaging of ophthalmic diseases for correlation analysis. For example, Zhong et al. and Huang et al. used AHBA for neuroimaging transcriptomic analysis of dynamic functional connectivity density and Voxel-Mirrored Homotopic Connectivity in patients with diabetic retinopathy, respectively (61, 62); Li et al. also utilized AHBA to explore ALFF values in different frequency bands in PACG patients (35). In addition, the dynamic molecular balance of the neurotransmitter receptor/transporter system may be the molecular basis mediating the remodeling of neural circuits, and the underlying regulatory mechanisms may stem from the chemical microenvironmental spatiotemporal-specific modulation of functional connectivity architectures at cortical hierarchical levels (63). Therefore, an in-depth analysis of the interaction between gene transcriptional regulation and neurotransmitter changes in dALFF will provide new perspectives for the elucidation of PACG pathomechanisms.

In the present study, we aimed to explore the dynamic changes in brain neural activity in PACG patients and its association with the brain genome in order to explore the underlying neural mechanisms and suggest possible early predictors in PACG patients. We propose that PACG patients exhibit significant abnormal dALFF alterations in whole brain regions, which are intrinsically associated with cortical gene expression patterns and neurotransmitter receptor/transporter distribution. In the present study, dALFF metrics of PACG patients were systematically analyzed to characterize their dynamic neural fluctuations from three sliding windows: 30TR, 50TR and 80TR, respectively. Then, the results of dALFF analysis from the 50TR sliding window were analyzed with the AHBA dataset by PLS to find the genes that were positively and negatively correlated with them. Multi-level probes were subsequently carried out, including biological pathway enrichment analysis, cell-specific expression profiling based on transcriptional decoding, and protein interaction network construction. Synchronously, under the 50TR time window parameter, we established the association mapping of neurotransmitter receptor/transporter brain region expression patterns with dALFF variations to explore the key roles of gene transcriptional regulation and neurotransmitter receptor/transporter changes in dALFF.

## Methods

### Participants

Participants 47 patients diagnosed with PACG were recruited from the same hospital, along with 46 carefully matched controls based on age, gender, and education. All experimental procedures were conducted in accordance with the Declaration of Helsinki, approved by the Ethics Committee of School of Ophthalmology and Optometry, Jiangxi Medical College, Nanchang University, and written informed consent was obtained from each subject.

Inclusion criteria for PACG patients were as follows: (1) bilateral narrow angle confirmed by atrial anguloscopy; (2) presence of visual field defects associated with glaucoma; (3) no history of glaucoma medication or surgical treatment; (4) no history of craniocerebral trauma; (5) no neurologic or psychiatric disorders; (6) availability of magnetic resonance imaging (no metallic implants, such as pacemakers); and (7) acceptance of the scans after the resolution of acute symptoms. Exclusion criteria: (1) Comorbid primary open-angle glaucoma (POAG) or secondary glaucoma; (2) Comorbid other ocular or systemic diseases affecting the visual pathway; (3) History of glaucoma or significant ocular disease; (4) History of antiglaucomatous surgery; (5) History of craniocerebral trauma; (6) Severe neurologic/psychiatric disorders; (7) Contraindications to magnetic resonance imaging examination; (8) Long-term use of neurologic medications affecting the brain’s function/structure; and (9) Presence of acute attack symptoms (e.g., eye pain) at the time of scanning; (10) Patients with cerebrovascular disease.

Inclusion criteria for the control group: (1) no organic lesions on ophthalmologic examination; (2) no history of craniocerebral trauma; (3) no neurologic/psychiatric disorders; (4) eligibility for magnetic resonance examination; and (5) demographic characteristics matching the PACG group. Exclusion criteria: (1) confirmed diagnosis of any ocular or systemic disorder; (2) severe refractive error (equivalent spherical lens ≥ ± 6.00 D); (3) history of craniocerebral trauma; (4) severe neurologic/psychiatric disorders; (5) contraindications to magnetic resonance examination; and (6) prolonged use of neurologic medications.

### fMRI data acquisition

A 750 T magnetic resonance imaging system manufactured by General Electric, United States, equipped with a novel 3.0-channel phased-array head coil, was used in this experiment. Functional magnetic resonance data of blood oxygen level dependent (BOLD) signals were acquired via a gradient echo planar imaging (EPI) sequence. Key imaging parameters included a repetition time (TR) of 2000 ms, a time to echo (TE) of 25 ms, a layer thickness of 3.0 mm, a layer spacing of 1.2 mm, a receiver matrix of 64 × 64, a radiofrequency flip angle of 90°, and an effective scanning field of 240 × 240 mm^2^. The voxel resolution was set to 3.6 × 3.6 × 3.6 mm^3^, totaling 35 layers axially covering the whole brain. Each scanning sequence consistently acquired 240 BOLD kinetic time points.

Before the experiment, participants were explicitly asked to keep both eyes open, maintain wakefulness, and avoid systematic thinking activities as much as possible. During data acquisition, the head was tightly immobilized with custom sponge padding to minimize potential motion artifacts in order to reduce noise. During the post-scanning phase, subjects instantly completed a standardized post-effects questionnaire, which was used to verify the degree of adherence to behavioral norms during the scanning process.

### fMRI data preprocessing

In this study, we used the Data Processing and Analysis Toolbox for Brain Imaging in conjunction with Statistical Parametric Mapping software^1^ on the MATLAB 2022b platform to standardize the preprocessing and analysis of functional magnetic resonance imaging (fMRI) data for standardized preprocessing analysis. The data preprocessing steps were as follows: (1) Convert the original image file format, and convert the DICOM raw data to the NIFTI common format; (2) Exclude the initial 10 time point data to eliminate the influence of magnetic field transient effects during the initialization stage of the MRI sequence; (3) Perform time alignment of adjacent slices, aligning the acquisition time of each slice with the midpoint of the repetition time (TR) to achieve multidimensional timing correction; (4) Apply a 24-parameter head motion correction model, set strict quality control standards (translation > 2 mm or rotation > 2°), and reject abnormal motion data; (5) Realize high-precision structural image segmentation based on the DARTEL algorithm to construct an individualized anatomical template, and then standardize the functional image to the MNI template space with a spatial resolution of 3 × 3 × 3 mm^3^; (6) Adopt a full-width-half-height (FWHM) of 6 mm 3D Gaussian kernel for spatial smoothing to optimize the signal-to-noise ratio of the signal; (7) Perform phase linear regression analysis to eliminate the low-frequency drift component in the time series; (8) Regression correction of the head movement parameters and the whole-brain averaged signals as orthogonal interference covariates to maximally control physiological noise interference.

### dALFF data processing

In the present study, the Time Dynamic Analysis built into DPABI v8.1 software was used for neuroimaging dynamic metrics measurements. Previous studies have shown that insufficient time window span exacerbates the time-series oscillatory characteristics of dynamic low-frequency amplitude fluctuation (dALFF) signals, where as an excessively long window length does not adequately reflect dynamic temporal changes in dALFF (64, 65). Therefore, the optimal selection of time window parameters constitutes one of the core challenges in the methodological study of dynamic functional magnetic resonance imaging, and sliding time-domain analysis techniques have a key methodological value in the quantitative assessment of the dynamic features of such spontaneous brain activities. To systematically eliminate the potential bias introduced by a single time-window configuration, the present study adopted a multiple time-window parameter validation strategy: 30TR (60 s), 50TR (100 s), and 80TR (160 s) with a 2TR sliding step for the full cohort dALFF calculation (45, 66–68). For the individual-level spatiotemporal dALFF feature parameters, we calculated the arithmetic mean and standard deviation of the time-varying signals of each voxel, and then deduced the distribution characteristics of its temporal coefficient of variation (CV = standard deviation/mean). The resulting dynamic coefficient of variation images were incorporated into the framework of subsequent between-group statistical analyses.

### Gene expression data processing

Data processing consisted of utilizing the AHBA database available at https://www.brainmap.org. This database provides gene expression profiles for six postmortem brains (male to female ratio: 5:1; mean age: 42.5 ± 13.4 years) covering 3,702 different spatial samples and measuring the expression levels of more than 20,000 genes. The raw dataset was subjected to full-scale standardized preprocessing, and established quality control protocols were strictly followed to ensure computational reproducibility. The AHBA dataset was processed according to Arnatkevic et al. (69). The six steps of preprocessing were as follows: (1) validation of probe-to-gene annotations using the Re-annotator toolkit (70); (2) filtering of probes (intensity-based filtering) to no more than background noise, excluding all samples from at least 50% of the participants; (3) probe selection, choosing the highest correlation with the RN-seq data; (4) assigning samples to wraps within a 2 mm Euclidean distance within the AAL90 atlas set; (5) Normalization of expression measurements using a scaled robust Sigmoid for each participant; (6) Gene set filtering based on differential stability. Since the AHBA dataset includes only two right-brain data, only the left brain was considered in our analyses (69). Therefore, the average of all samples in the regions was calculated to obtain a matrix of transcript level values (90 regions × 10,027 gene expression levels). We applied partial least squares (PLS) regression to model multivariate associations between dALFF (time window = 50 TRs) and 15,633 high-quality gene features, where the PLS principal component characterizes the optimal linear combination of gene expression profiles and neural activity dynamics with maximum covariance.

We performed correlation analyses using PLS1 and PLS2 methods, respectively; however, unfortunately in the PLS1 analyses, the *p*-values of most results exceeded the threshold of 0.05 after FDR correction, and therefore we only used PLS2 results for exploration in subsequent studies. The 2nd PLS regression component (PLS2) was highly correlated with regional differences in dALFF. Spatial alignment tests (10,000 trials) were used to verify whether PLS2 was statistically significant. To estimate the variability of each gene in PLS2, a bootstrap method was used to generate Z-values by calculating the weight of each gene with respect to the standardized bootstrap error for that gene and ranking the genes according to their contribution to PLS2. Significant genes with positive (PLS+) and negative (PLS–) weights of FDR-corrected 5‰ were screened.

### Enrichment analysis

Enrichment analysis was performed for genes that were significantly positively and negatively correlated with their dALFF values in the 50TR window, respectively. This analysis utilized the DAVID functional annotation bioinformatics microarray analysis platform to explore biological functions. Gene ontology (GO) terms including biological process (BP) were used to assess biological functions. In addition, related biological pathways were investigated with a focus on the Kyoto Encyclopedia of the Genome (KEGG) pathway. All enrichment analysis maps were generated using Metascape and are available at https://metascape.org/gp/. Enrichment pathways were derived by entering PLS1 + (Z > 5) or PLS1- (Z < − 5) on the Metascape website, followed by a significance threshold of 5%.

### Specific expression analysis

Specific expression analysis was performed for genes significantly positively and negatively correlated within the TR50 window of dALFF. Analyses were performed using the CSEA tool accessible at http://doughertytools.wustl.edu/CSEAtool.html and focused on identifying genes that showed over-representation in different cell types, brain regions and developmental stages. Cell-specific expression analysis was performed to demonstrate the unique expression of the genes of interest in various cell types; brain-specific expression analysis highlighted their specific expression patterns in different brain regions; and time-specific expression analysis further elucidated their differential expression in developmental stages and brain regions. Thresholds for probability of specificity index (pSI) were 0.05, 0.01, 0.001, and 0.0001.

### Proteins and protein interactions

Protein–protein interaction (PPI) analysis was performed on significantly positively and negatively correlated genes within the TR50 window of dALFF. This analysis was performed using STRING v11.0^2^ to investigate the potential formation of PPI networks between genes associated with altered brain function. In addition, the most highly connected genes were selected and used to map their spatiotemporal and temporal expression patterns through the Human Brain Transcriptome Database.^3^

### Neurotransmitter receptors/transporters distribution maps

The neurotransmitter density map is derived from positron emission tomography (PET) images of over 1,200 healthy individuals, encompassing 19 distinct neurotransmitter receptors and transport proteins from nine different neurotransmitter systems. Twenty different neurotransmitter receptor/transmitter whole-brain density profiles were selected from previous *in vivo* molecular imaging studies, including 5-HT1AR, 5-HT1BR, 5-HT2AR, 5-HT4R, 5-HT6R, 5-HTT, α4β2, CB1R, D1R, D2R, DAT, FDOPA, GABAAR, H3R, M1R, mGluR5, MU, NAT, NMDA and VAChT^4^ (71–75). Subsequently, receptor/transporter density values were extracted from each positron emission tomography atlas and averaged over 90 regions according to anatomical autolabeling atlas (AAL). For the 50 TR window of dALFF values, we performed a two-sample t-test. Therefore, we extracted the t-values and averaged them in the same way as the receptor/transporter density procedure. Finally, we performed a correlation analysis to investigate the relationship between *t*-values and neurotransmitter receptor/transporter density after the two-sample test.

### Statistical analysis

**Figure 1:**
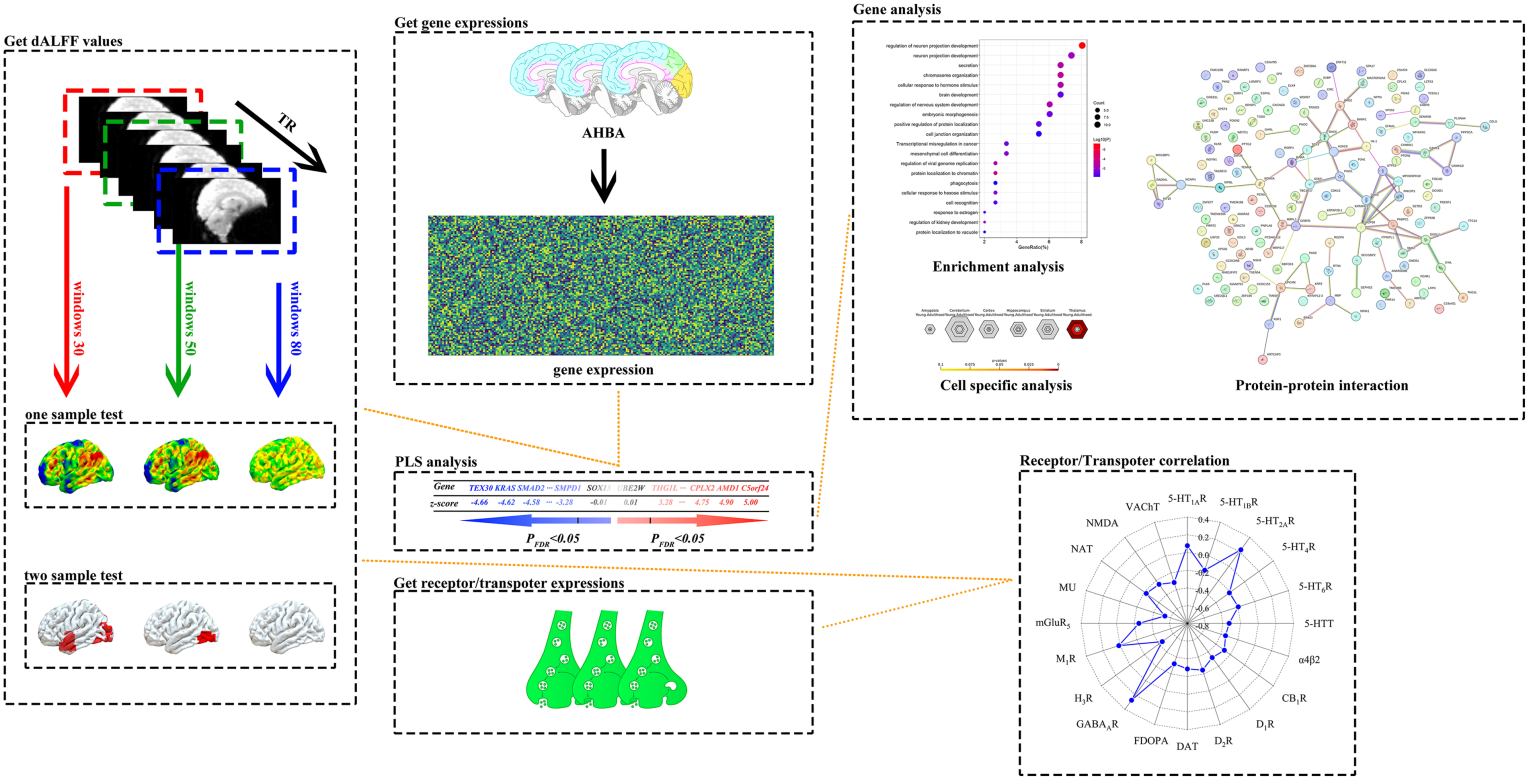
A systematic data analysis process was used in this study. The study first implemented one-sample t-tests and two-sample t-tests for dALFF values calculated in 30TR, 50TR, and 80TR time window conditions for PACG patients and HC subjects, respectively, and used Gaussian Random Field Theory (GRF) multiple comparisons correction method (*p* < 0.005 at the voxel level, and *p* < 0.01 at the clump level) to statistically correct the results to ensure reliability. Subsequently, under the 50TR sliding window condition, we performed PLS regression analysis of the t-statistics of significantly different dALFF values obtained from the two-sample t-test with the multigene expression values of each brain region in the Allen Human Brain Atlas (AHBA), and successfully extracted the genome-wide set of genes characterizing the associations with positively correlated (PLS+) and negatively correlated (PLS-) patterns. The resulting genes were subjected to enrichment analysis, specific expression analysis and protein–protein interaction analysis. In addition, the study innovatively explored the above patterns of association between dALFF differential t-values and the spatial distribution of neurotransmitter receptor/transporter expression levels between brain regions. dALFF, dynamic low-frequency fluctuation amplitude; TR, repetition time; PLS, partial least squares; PACG, primary angle-closure glaucoma; HC, healthy control. Parts of this figure were drawn by using pictures from Biovisart (https://biovisart.com.cn).

We statistically analyzed the clinical characteristics of the two groups of patients using the Statistical Package for the Social Sciences (SPSS)26 software from IBM, Armonk, NY, United States. Between-group differences in dALFF values for 30TR, 50TR, and 80TR were assessed by two-sample t test assessed with age, gender and head movement as covariates. Multiple comparisons were corrected using the Gaussian Random Field (GRF) method, with significance at the voxel level set at *p* < 0.005 (two-tailed) and at the cluster level at *p* < 0.01. The correlation of neurotransmitters/transporters with the dALFF values in the 50TR window was set at 0.05 (two-tailed) using a threshold of significance and corrected for FDR. All procedures are shown in Figure 1.

## Results

### Demographics and disease characteristics

In terms of demographic characteristics, the PACG and HC groups did not present statistically significant differences in gender (*p* = 0.45), age (*p* = 0.86), and years of education (*p* = 0.38). Notably, compared to the HC group, the PACG group presented statistically significant differences in key visual function indicators such as binocular best-corrected visual acuity (BCVA) (*p* < 0.001) and intraocular pressure (IOP) (*p* < 0.001; Table 1).

**Table 1:** Demographics and visual measurements between two groups.

Condition	PACG group	HC group	*t/ꭓ*^2^value	*p* value
Gender (male/female)	26/21	29/17	0.57	0.45
Age (years)	54.53 ± 10.03	55.91 ± 10.00	−0.184	0.86
Education	14.86 ± 1.56	15.14 ± 1.51	−0.886	0.38
BCVA-OD	0.51 ± 0.17	1.11 ± 0.06	−22.15	<0.001*
BCVA-OS	0.49 ± 0.15	1.11 ± 0.06	−26.44	<0.001*
IOP-OD	48.42 ± 1.78	14.53 ± 1.06	111.24	<0.001*
IOP-OS	48.02 ± 1.53	15.10 ± 1.20	115.04	<0.001*

BCVA, best corrected visual acuity; IOP, intraocular pressure; OD, oculus dexter; OS, oculus sinister. * indicate *p* < 0.001.

### Values of dALFF in different windows

**Figure 2:**
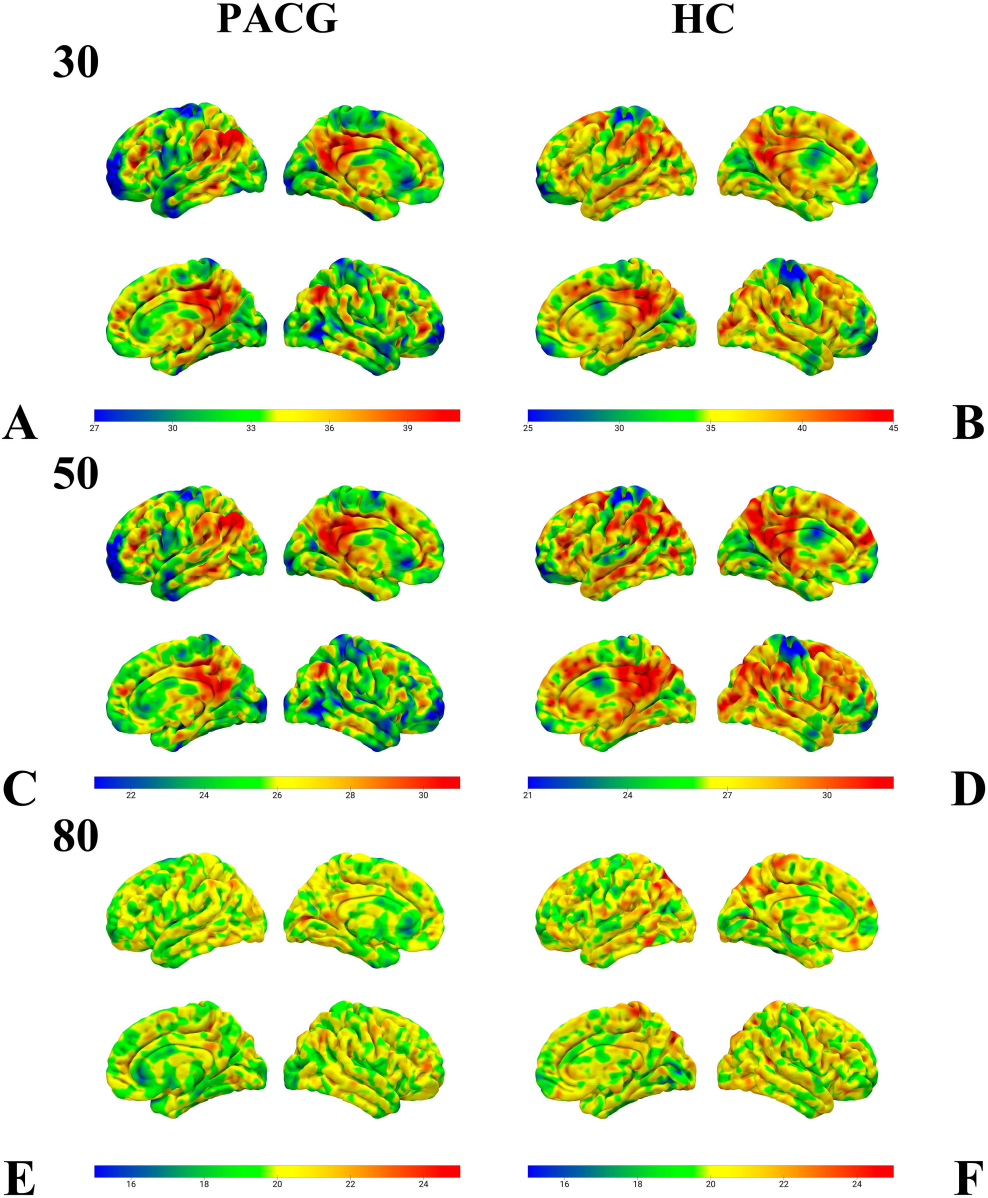
This figure presents the results of the one-sample test of dALFF values in the sliding windows of 30TR **(A,B)**, 50TR **(C,D)**, and 80TR **(E,F)** for the PACG group and HC group, respectively. Notably, despite the differences in time window parameters, both the PACG and HC groups showed very similar spatial distribution patterns within their respective groups. dALFF, amplitude of dynamic low-frequency fluctuations; TR, repetition time; PACG, primary closed-angle glaucoma; HC, healthy control.

Performing a one-sample test for the PACG and HC groups in 30TR, 50TR, and 80TR sliding windows, respectively, we found that the spatial distribution of dALFF at all three temporal resolutions showed a significant consistency within the groups (Figure 2).

**Figure 3:**
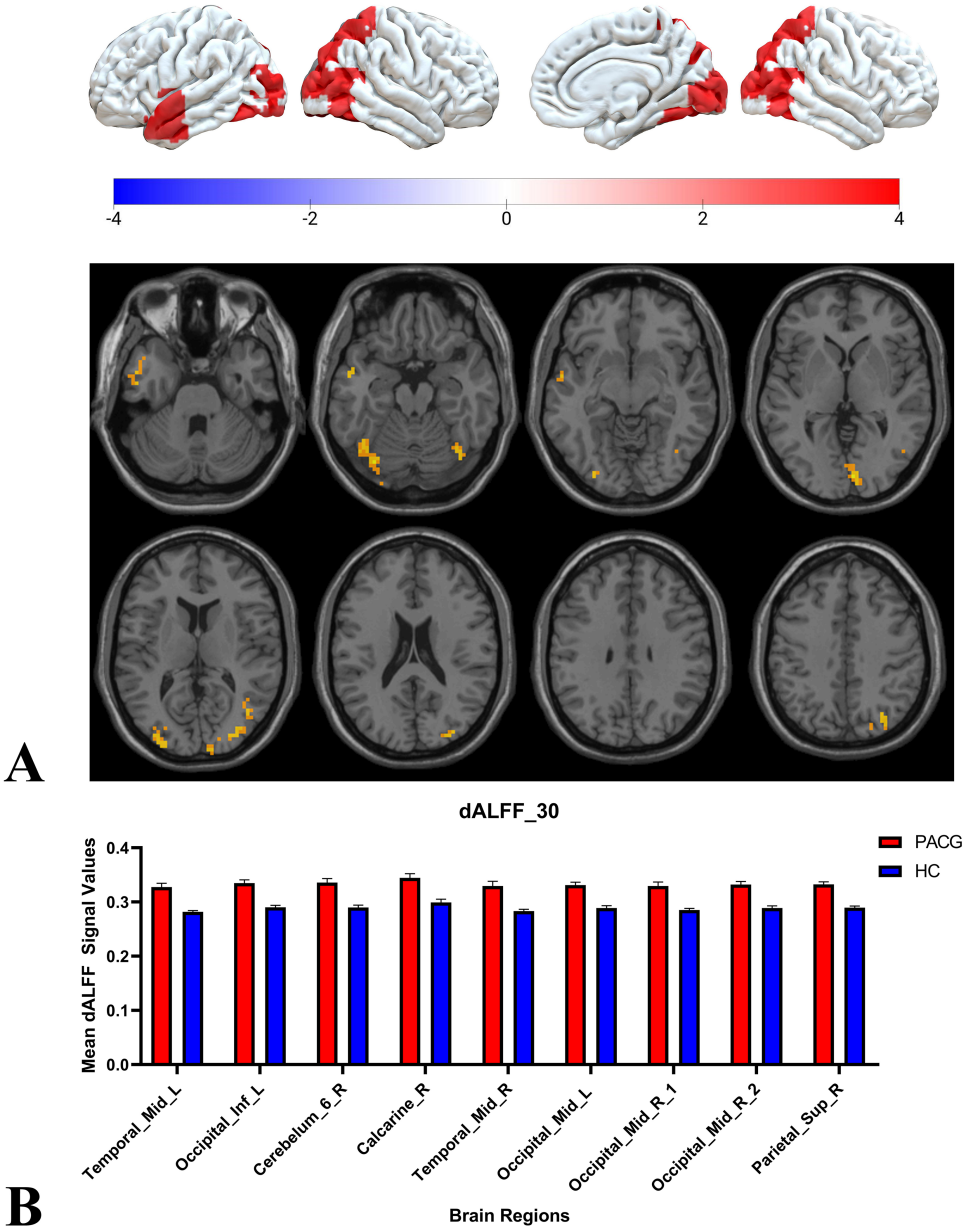
This figure represents the two-sample *t*-test results of dALFF values in the PACG and HC groups in a 30 TR sliding window. The results indicate that the PACG group had extensive alterations in dALFF values compared to the HC group and all of them showed elevated values. temporal_Mid_L, left middle temporal gyrus; Occipital_Inf_L, left suboccipital gyrus; Cerebelum_6_R, right Cerebellum Superior; Calcarine_R, right perisylvian cortex; Temporal_Mid_R, right middle temporal gyrus; Occipital_Mid_L, left middle occipital gyrus; Occipital_Mid_R, right middle occipital gyrus; Parietal_Sup_R, right parietal inferior marginal angular gyrus; dALFF, dynamic low-frequency fluctuation amplitude; TR, repetition time; PACG, primary angle-closure glaucoma; HC, healthy control.

**Figure 4:**
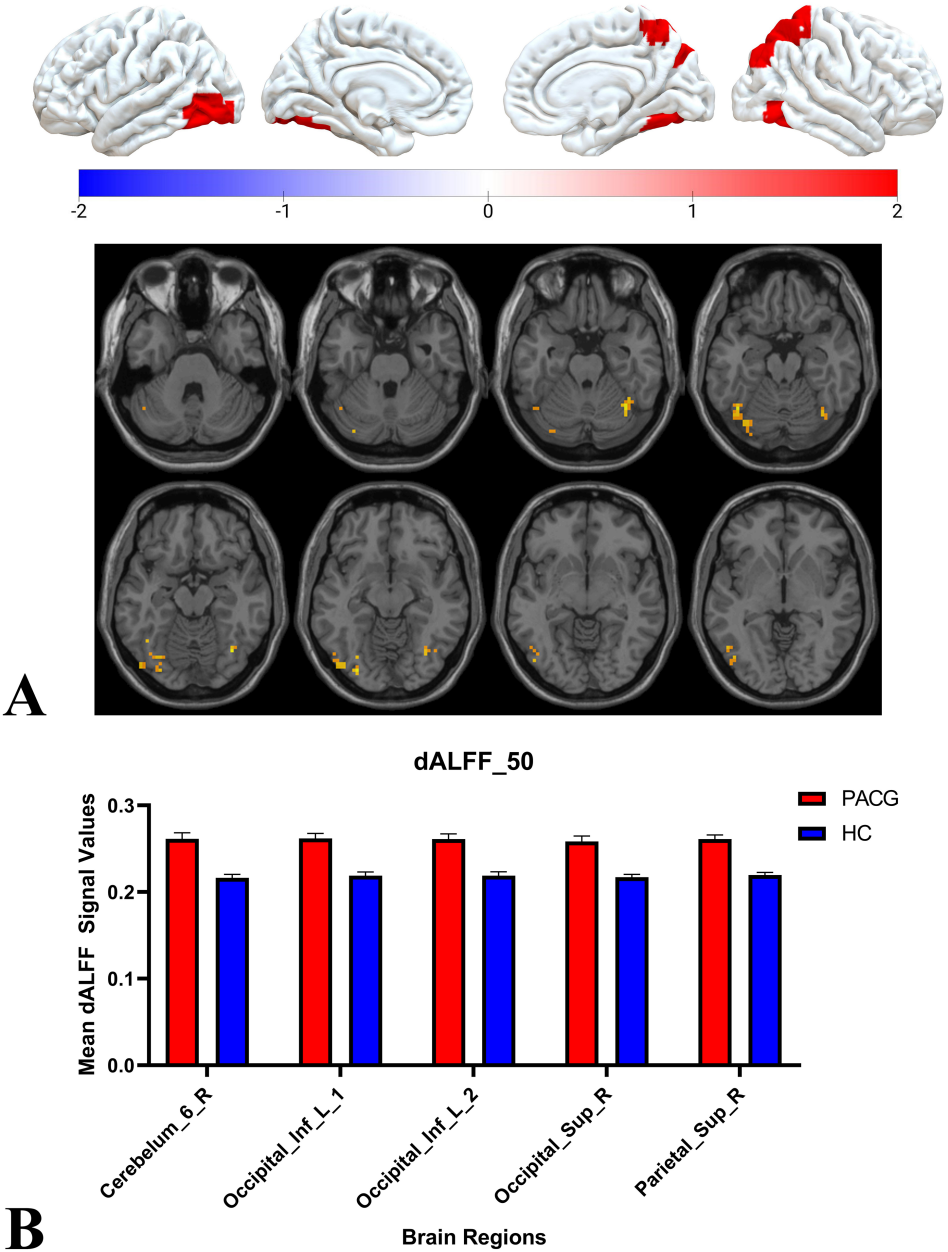
This figure represents the results of the two-sample t-test for dALFF values in the PACG and HC groups in the 50TR sliding window. The results show that compared to the HC group, the PACG group had altered dALFF values in a wide range of brain regions mainly in the occipital lobe and all of them showed elevated dALFF values. occipital_Inf_L, left suboccipital gyrus; Cerebelum_6_R, right Cerebellum Superior; Occipital_Sup_R, right superior occipital gyrus; Parietal_Sup_R, right inferior parietal marginal angle gyrus; dALFF, dynamic low-frequency fluctuation amplitude; TR, repetition time; PACG, primary angle-closure glaucoma; HC, healthy control.

**Figure 5:**
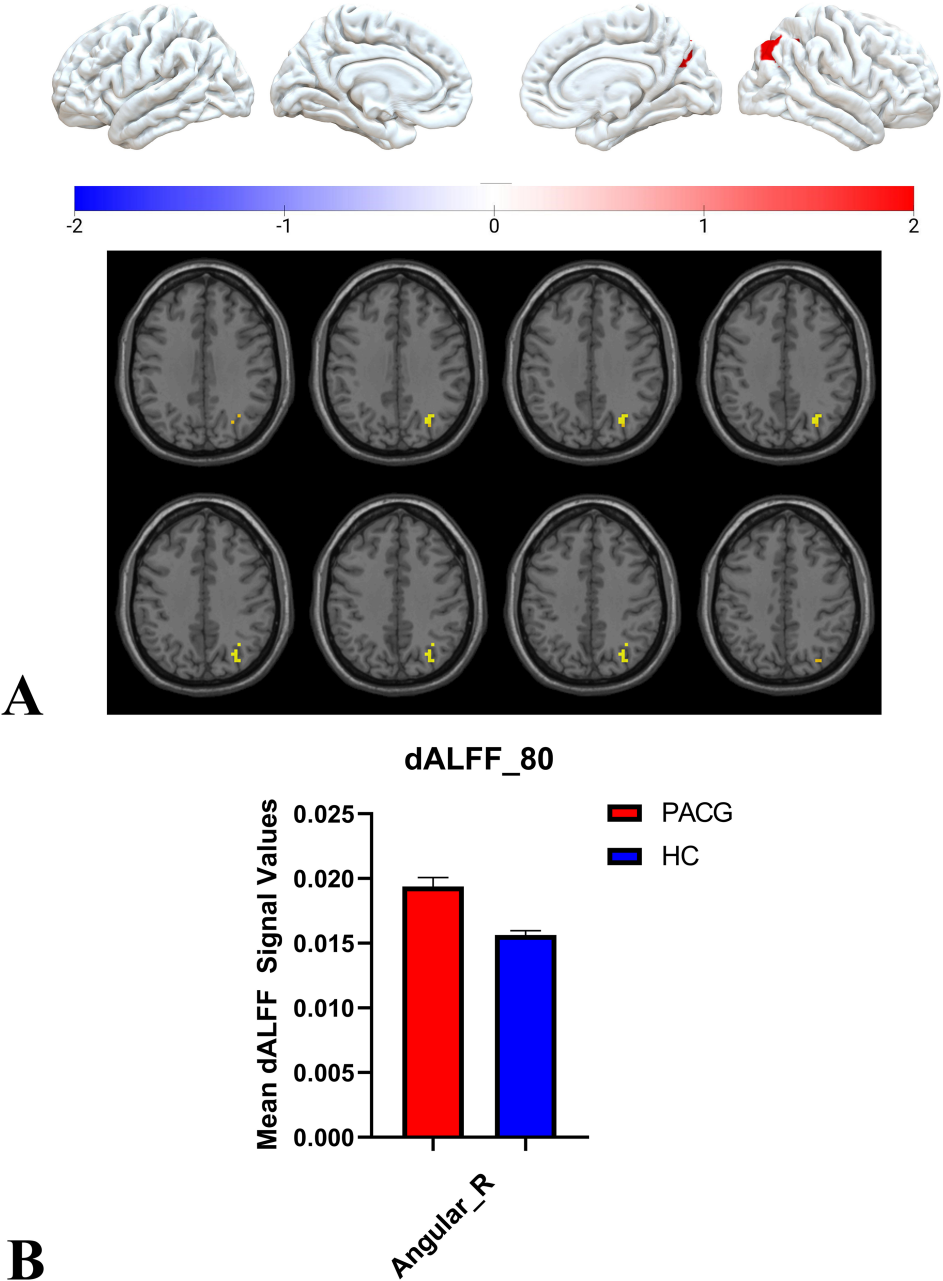
This figure represents the two-sample t-test results of the dALFF values in the PACG and HC groups in the 80 TR sliding window. The results indicate that the dALFF values of the right angular gyrus were altered and elevated in the PACG group compared with the HC group. angular_R, right angular gyrus; dALFF, amplitude of dynamic low-frequency fluctuations; TR, repetition time; PACG, primary angle-closure glaucoma; HC, healthy control.

Two-sample t-tests showed that under 30TR, 50TR, and 80TR dynamic window conditions, PACG patients presented significantly higher dALFF amplitudes in all scan states compared with healthy controls. in the 30TR sliding window, the regions of significant change were the left middle temporal gyrus (Temporal_Mid_L), the left suboccipital gyrus (Occipital_Inf_L), right Cerebellum Superior (Cerebelum_6_R), right periaqueductal cortex (Calcarine_R), right middle temporal gyrus (Temporal_Mid_R), and left middle occipital gyrus (Occipital_Mid_L), right middle occipital gyrus (Occipital_Mid_R), right subparietal marginal angular gyrus (Parietal_Sup_R) (Table 2; Figure 3A); the mean values of dALFF in 30TR between two groups was showed in Figure 3B, in the 50TR sliding window, the abnormally altered brain regions were right Cerebellum Superior (Cerebelum_ 6_R), left suboccipital gyrus (Occipital_Inf_L), right supraoccipital gyrus (Occipital_Sup_R), and right parietal submarginal angular gyrus (Parietal_Sup_R) (Table 2; Figure 4A); the mean values of dALFF in 50TR between two groups was showed in Figure 4B, and in the 80TR sliding window, the abnormally altered brain region was the right angular gyrus (Angular_R) (Table 2; Figure 5A). The mean values of dALFF in 80TR between two groups was showed in Figure 5B.

**Table 2:** Results of the dALFF two-sample test for the PACG and HC groups under different sliding windows.

Condition	Voxel	MNI coordinate			peak *t*-value
		*x*	*y*	*z*	
Window size of 30 TR and sliding step of 2 TR					
Temporal_Mid_L	43	−51	0	−18	3.9026
Occipital_Inf_L	68	−30	−84	−12	4.6844
Cerebelum_6_R	37	36	−60	−21	5.0446
Calcarine_R	34	6	−87	0	4.3162
Temporal_Mid_R	38	45	−72	9	4.2708
Occipital_Mid_L	46	−33	−90	9	4.2042
Occipital_Mid_R_1	67	33	−84	9	4.5182
Occipital_Mid_R_2	36	33	−78	36	4.2371
Parietal_Sup_R	55	24	−69	63	4.6455
Window size of 50 TR and sliding step of 2 TR					
Cerebelum_6_R	38	36	−60	−21	4.7551
Occipital_Inf_L_1	55	−30	−84	−12	4.4546
Occipital_Inf_L_2	31	−51	−69	−9	4.8652
Occipital_Sup_R	34	30	−75	42	4.2786
Parietal_Sup_R	54	24	−69	63	4.8628
Window size of 80 TR and sliding step of 2 TR					
Angular_R	18	33	−60	39	3.6643

Temporal_Mid_L, left middle temporal gyrus; Occipital_Inf_L, left suboccipital gyrus; Cerebellum_6_R, right Cerebellum Superior; Calcarine_R, right peri-talar fissure cortex; Temporal_Mid_R, right middle temporal gyrus; Occipital_Mid_L, left middle occipital gyrus; Occipital_Mid_R, right middle occipital gyrus; Parietal_Sup_R, right subparietal marginal angular gyrus; Occipital_Sup_R, right superior occipital gyrus; Angular_R, right angular gyrus; dALFF, dynamic low-frequency fluctuation amplitude; TR, repetition time; PACG, primary angle-closure glaucoma; HC, healthy control; MNI, Montreal Neurological Institute.

### 50TR sliding window dALFF values correlate with cortical gene expression

**Figure 6:**
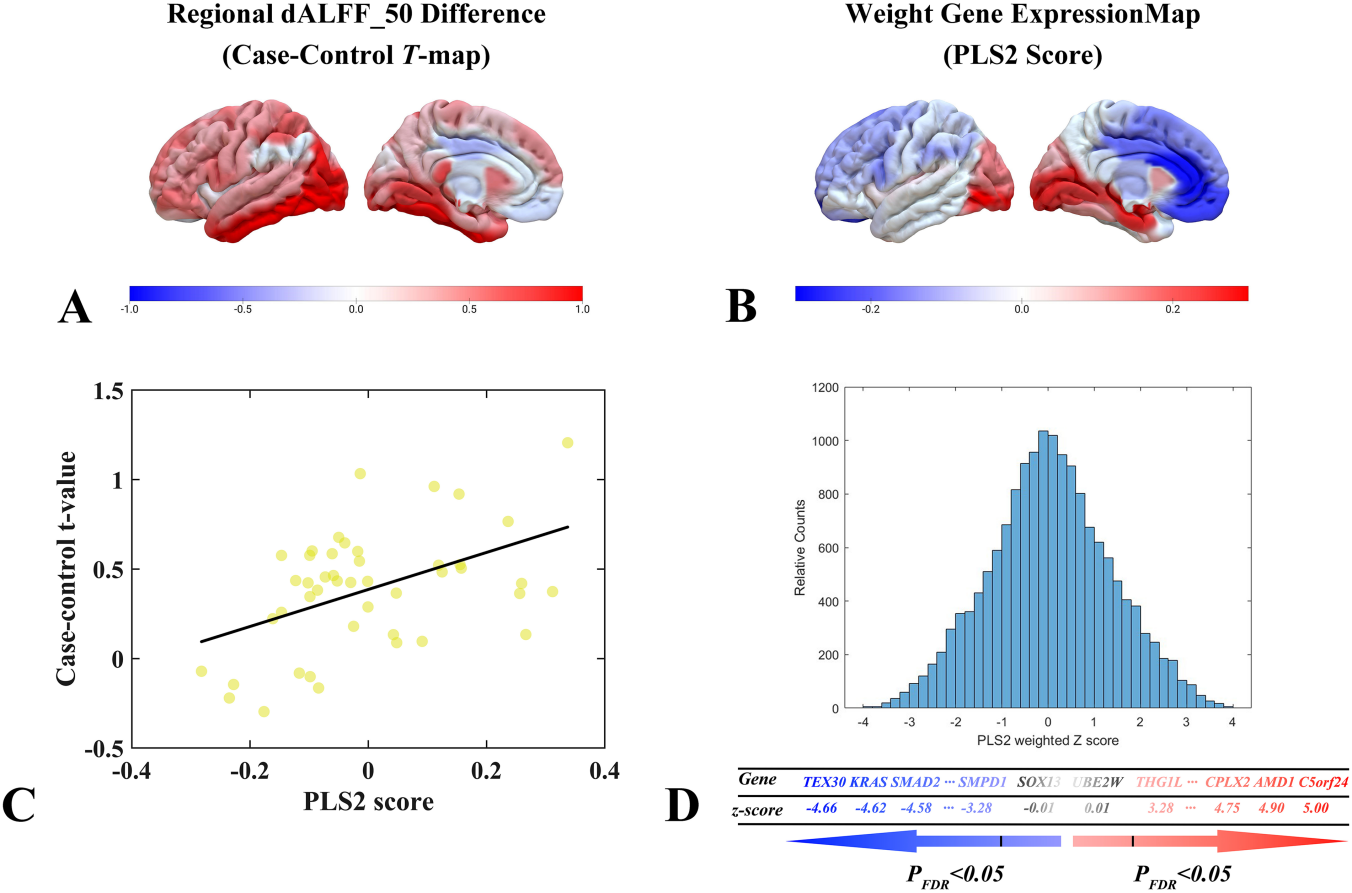
dALFF values under the 50TR sliding window correlate with cortical gene expression. **(A)** Distribution of brain regions with two-sample test t-values for dALFF values in the 50TR sliding window in the left hemisphere region. **(B)** Three-dimensional spatial distribution of weighted gene expression profiles constructed by the left hemisphere regional PLS2 algorithm in the left hemisphere cortex. **(C)** Scatterplot of regional PLS2 scores (weighted sum of 15,633 gene expression scores) and changes in dALFF values (Pearson’s r = 0.3742, pspin = 0.0087) under a 50TR sliding window. **(D)** Schematic diagram of the process of performing PLS + versus PLS- gene screening. dALFF, dynamic low-frequency fluctuation amplitude; PLS, partial least squares; PACG, primary angle-closure glaucoma; HC, healthy control.

In this study, the PLS system was used to explore the pattern of association between the dALFF feature of the 50TR sliding window (Figure 6A) and genome-wide gene expression profiles of 15,633 genes. In the discovery cohort, PLS2 explained 51.37% of the cross-modal variance. The distribution of PLS2-weighted profiles reflected the anterior–posterior gradient of gene expression (Figure 6B). Specifically, gene expression profiles weighted by PLS2 correlated significantly with case–control differential t-value profiles (Pearson’s r = 0.3742, pspin = 0.0087; Figure 6C). A total of 340 genes were identified as significantly associated with the dynamic features of dALFF after correction for strict multiple comparisons (*p* < 0.05, FDR correction). Among them, 155 were PLS + genes and 185 were PLS- genes (Figure 6D).

### Enrichment analysis

**Figure 7:**
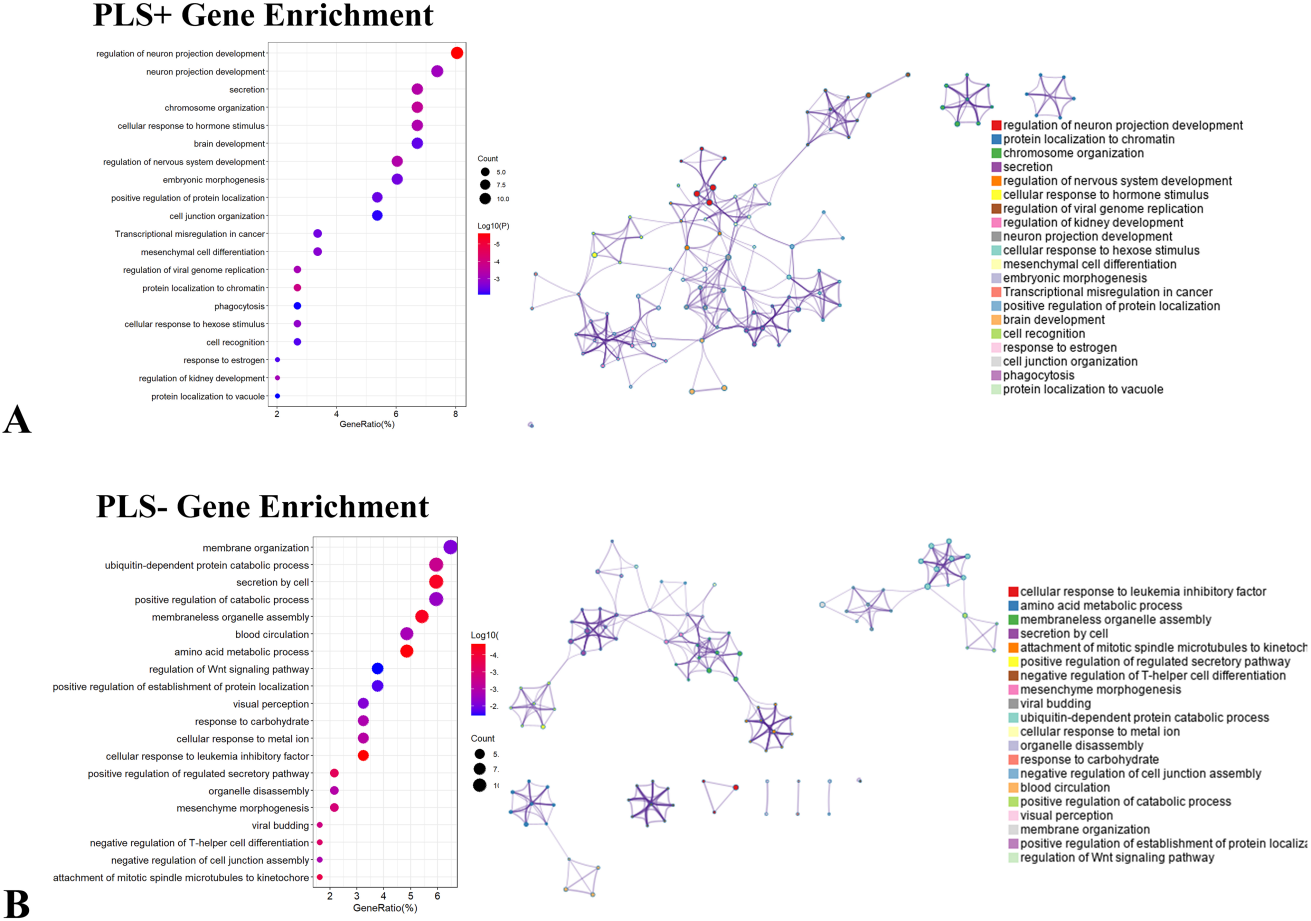
Enrichment analysis of genes associated with dALFF values PLS + and PLS- under the 50TR sliding window. **(A)** Ontology terms for PLS + genes (*p* < 0.05, FDR-corrected). **(B)** Ontological terms for PLS- genes (*p* < 0.05, FDR-corrected). dALFF, dynamic low-frequency fluctuation amplitude; PLS, partial least squares; PACG, primary angle-closure glaucoma; HC, healthy control.

We performed a systematic enrichment analysis of the finely screened PLS + and PLS- gene clusters to reveal their functional characteristics. We systematically compared the PLS + genome with the Gene Ontology bioprocess and KEGG pathway databases. Ontology terms for PLS + genes (*p* < 0.05, FDR-corrected) (Figure 7A). Notably, the top 20 significantly enriched GO biological processes (e.g., “regulation of neuron projection development,” “neuron projection development,” and “secretion”) exhibited significant associations, but KEGG pathway analysis did not detect a PLS-gene showed significant enrichment in GO processes, including “membrane organization,” “ubiquitin-dependent protein catabolic process” and “secretion by cell,” but KEGG pathway analysis also did not show significant correlation (Figure 7B).

### Specific expression analysis

**Figure 8:**
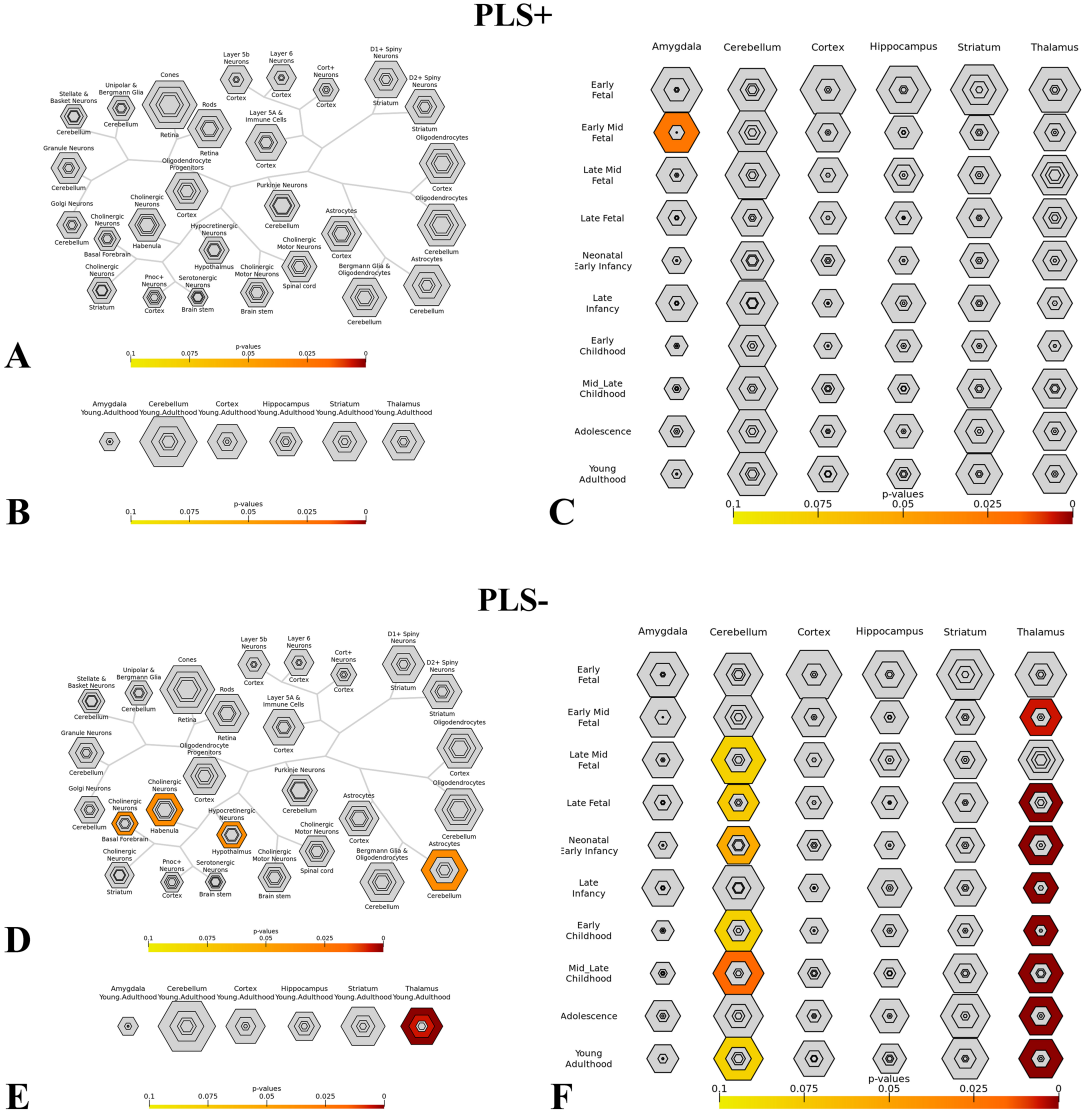
This figure shows the specific expression analysis of genes associated with dALFF values PLS + and PLS- under the 50TR sliding window. **(A,D)** Cell-specific expression analysis. **(B,E)** Brain-specific expression analysis. **(C,F)** Time-specific expression analysis. (Thresholds were set at *p* = 0.05, *p* = 0.01, *p* = 0.001 and *p* = 0.0001). dALFF, dynamic low frequency fluctuation amplitude; PLS, partial least square.

For genes associated with dALFF values PLS + and PLS- under the 50TR sliding window, we performed cell-specific expression analysis, brain-specific expression analysis, and time-specific expression analysis. Among PLS + genes, Cellspecific expression analysis (Figure 8A), brain-specific expression analysis (Figure 8B). Among PLS + genes, there was significant expression in the amygdala in early fetal life (Figure 8C); for PLS- genes, there was cell-specific expression in cholinergic neurons of the basal forebrain and rhabenula, and in Hypocretinergic Neurons of the Hypothalmus with astrocytes of the cerebellum expression was significant (Figure 8D), in brain-specific expression in the thalamus (Figure 8E), and in time-specific expression in the cerebellum of mid-late childhood versus the thalamus of early and late fetal, neonatal, childhood, adolescence, and early adulthood (Figure 8F).

### Protein–protein interaction analysis

**Figure 9:**
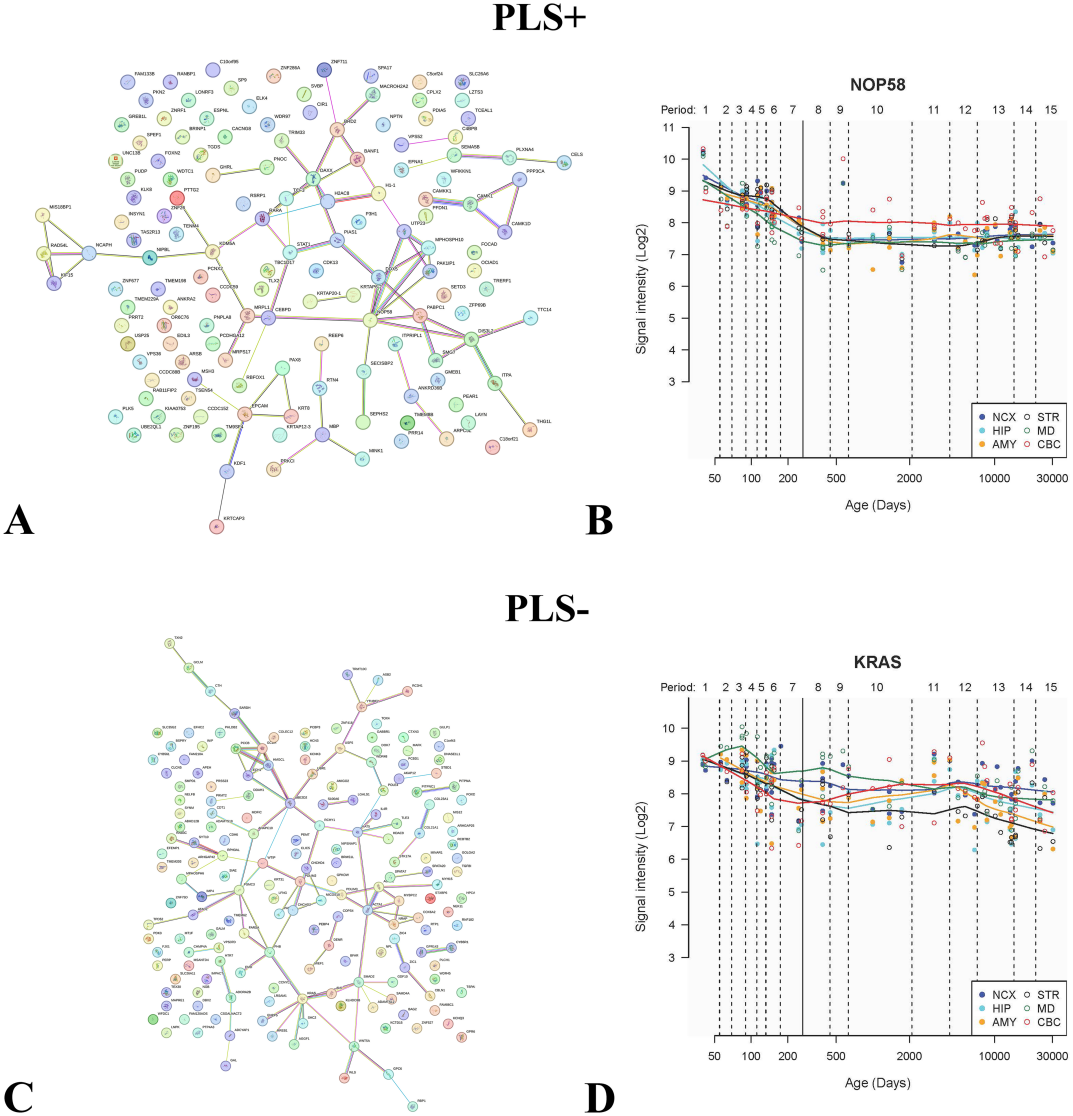
This graph shows the PPI analysis of genes associated with dALFF values PLS + and PLS- under the 50TR sliding window. **(A,C)** shows the PPI analysis plot for all genes, where the wired connections are the genes that make up the PPI network. **(B,D)** indicates the spatiotemporal specific expression analysis maps of the selected genes with the highest degree values. dALFF, dynamic low frequency fluctuation amplitude; PLS, partial least squares; PPI, protein–protein interaction.

We performed PPI analysis of PLS + -associated genes and PLS-associated genes separately and selected the genes with the highest degree value for spatiotemporal specific expression analysis. Among PLS + genes, a total of 64 genes formed an interconnected PPI network (Figure 9A), among which NOP58 (degree value = 8) was selected for spatiotemporal specific expression analysis (Figure 9B); among PLS- genes, a total of 89 genes formed an interconnected PPI network (Figure 9C), among which KRAS (degree value = 8) was analyzed for spatiotemporal specific expression (Figure 9D).

### Neurotransmitter receptor/transporter correlation analysis

**Figure 10:**
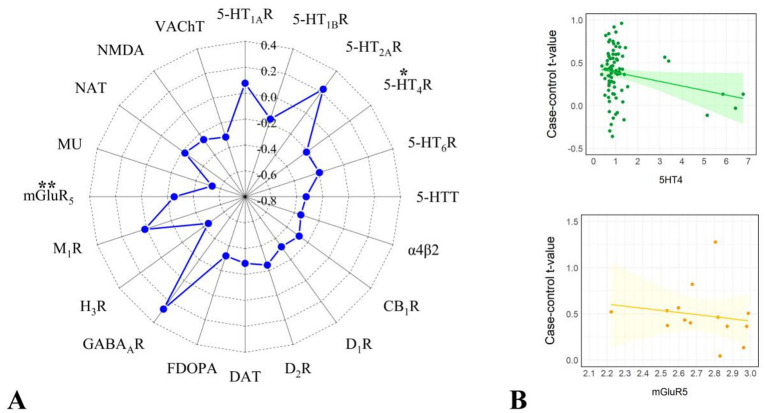
Results of correlation analysis of dALFF values with receptors/transporters under 50TR sliding window. **(A)** Indicates the radar plot of r-value of correlation analysis of each receptor/transporter with dALFF values, *indicates significant results (*p* < 0.05, pspin<0.05), **indicates significant results (*p* < 0.01, pspin<0.01). **(B)** Graphs indicating the correlation analysis of each receptor/transporter with dALFF values with significant results.

We correlated the dALFF values under the 50TR sliding window with the expression matrix of neurotransmitter receptors/transporters obtained. The results revealed significant correlations for 2 neurotransmitter receptors/transporters, 5-hydroxytryptamine type 4 receptor (5-HT4R) and metabotropic glutamate receptor 5 (mGlu5R) (Figure 10; Table 3).

**Table 3:** Results of correlation analysis of abnormal dALFF values in 50 TR with significant receptors/transporters.

Receptor/Transporter	*p* value	*p_spin_* value	*r* value
5HT4R	0.03	0.01	0.23
mGluR5R	<0.01	0.01	0.27

## Discussion

In this study, dALFF was analyzed in PACG patients and healthy controls using the 30TR, 50TR and 80TR sliding window methods, and it was found that compared to the HC group, PACG patients showed more significant dALFF enhancement in all three time windows analyzed, and this functional alteration was mainly focused on neuroanatomical structures such as the occipital cortex, the upper cerebellar region and the angular gyrus. A total of 340 genes were identified to show significant associations with neuroimaging features by PLS correlation analysis, including 155 PLS + genes and 185 PLS- genes, and quantitative correlation maps between gene expression and brain function abnormalities were established. Gene ontology enrichment analysis revealed that these genes were significantly enriched in key biological processes such as “neuronal projection developmental regulation” (GO:0031175) and “biofilm organization” (GO:0061024), suggesting that they are involved in the molecular mechanisms of neural circuit remodeling and cell membrane stability. Time-specific expression analysis showed that PLS-associated genes showed significant enrichment (FDR < 0.05) at several key stages of thalamic development, which revealed the potential role of abnormal thalamic development in the pathomechanism of PACG from an ontogenetic point of view. Ppi analysis indicated that most of the genes associated with PACG were involved in the composition of the ppi network, of which the pivotal genes were NOP58 and KRAS. correlation analysis with neurotransmitter receptors/transporters revealed that 5-HT4R and mGlu5R showed significant correlation. These multi-omics findings systematically elucidated the possible molecular regulatory network of PACG, and constructed a multi-scale pathology model from gene expression disorders to clinical phenotypes by integrating brain functional imaging features, genetic regulatory elements and neurotransmitter system abnormalities.

The occipital lobe, as a core brain area for visual information processing, has the ability to integrate visual functions at multiple levels. Its primary visual cortex (area V1) is responsible for receiving and parsing visual signals transmitted from the retina and completing the recognition of basic features such as light, shape, and color (76), whereas the higher visual cortex (e.g., mid-occipital region) is involved in complex visual tasks including object recognition, spatial localization, and facial emotion perception, and plays an important role in visual memory and dream generation (77, 78). In PACG, elevated IOP due to atrial angle obstruction not only directly damages optic ganglion cells, but may also indirectly affect occipital lobe function by disrupting visual conduction pathways (48, 79). For example, the occipital cortex of PACG patients showed significant functional reorganization characteristics (34). As found in this study, patients with PACG showed a wide range of abnormally elevated occipital dALFF values, and this elevation can be interpreted from a variety of layers. At the level of neural mechanisms, the occipital lobe may be involved in the pathological process of PACG through compensatory mechanisms: reduced visual input triggers enhanced compensatory neural activity, such as the right inferior occipital lobe that may compensate for peripheral visual field deficits by enhancing face processing (33, 34). Further, high dALFF values in the right inferior occipital lobe may reflect local neuronal hyper-synchronization activity, a compensatory mechanism that alleviates dysfunction associated with visual field deficits by enhancing complex visual information processing (34, 80). At the clinical level, changes in these dynamic parameters show a correlation with the degree of visual impairment, e.g., the magnitude of elevation of right inferior occipital ALFF is negatively correlated with the thinning of the optic nerve fiber layer (r = −0.456, *p* < 0.01) (34), suggesting that it can be used as an objective indicator for noninvasive assessment of disease severity. In addition, the development of cognitive complications in PACG may also involve functional reorganization of the occipital lobe, which has been found to correlate with altered functional connectivity in attention and executive function networks (77, 81). Genomics studies have further revealed that PACG susceptibility genes such as COL11A1 may directly influence the metabolic activity of the occipital lobe, providing new directions for understanding the genetic basis of central compensatory mechanisms (79, 82, 83). In summary, the functional remodeling of the occipital lobe in PACG is the result of the interaction between visual pathway damage and central compensation. The dynamic changes in its dALFF values not only reflect the pathophysiological characteristics of the disease, but also provide a theoretical basis for the development of novel therapeutic strategies targeting visual-neuroprotection.

The superior cerebellum consists of the anterior, superior-posterior, middle and anterior-superior portions of the earth, which regulate postural coordination, motor planning, sensory integration and balance functions, respectively, and its deeper nuclei such as the dentate nucleus and the raphe nucleus are widely connected to the central nervous system through the midbrain peduncle, and together they are involved in motor execution and cognitive-emotional regulation (84–87). Studies have shown that in addition to the anatomical factors of mechanical obstruction of atrial water drainage, the development of PACG may have an indirect interactive effect with abnormalities in the cerebellar functional network (48, 88, 89). Specifically, the anterior cerebellar lobes and the earthworms influence the activity of brainstem autonomic nuclei through specific neural pathways, and their dysfunctional connectivity may interfere with atrial water dynamic homeostasis (85, 86, 88). In this study, we found that dALFF in the upper cerebellum of PACG patients showed abnormally elevated dALFF, which may be mainly due to two reasons: on the one hand, the enhanced compensatory dynamic activity of neurons may partially alleviate the sensory-motor dysfunction caused by visual injury (34, 88), for example, the MRI evidence showed that neuronal synchronization and local coherence (ReHo) in their specific regions of the cerebellum were significantly enhanced, suggesting that the compensatory neural network remodeling in response to visual input deficits (34, 88, 90, 91); on the other hand, chronic IOP abnormalities may induce alterations in cerebellar-cortical synaptic plasticity, resulting in an abnormal functional state of high energy consumption and low efficiency (33, 92). In addition, the cerebellar dALFF dynamic parameter combined with a machine learning model can differentiate PACG patients with high precision, and its degree of abnormality is significantly correlated with retinal nerve fiber layer thinning, suggesting the potential value of this index as a biomarker for disease progression assessment (33, 88). Nevertheless, in the future, the integration of multimodal imaging and molecular genetics data is still needed to systematically elucidate the precise mechanism of the cerebellar network’s role in the regulation of atrial fluid dynamics and neurodegenerative processes.

The angular gyrus is an important brain region in the posterior parietal lobe, located at the junction of the temporal, parietal, and occipital lobes, and its functions are characterized by a high degree of multimodal integration (93, 94), encompassing, e.g., language processing (95, 96), memory integration and retrieval (97, 98), mathematical spatial and cognitive (99, 100), and self-awareness and social cognition (94, 96). The main reason for the abnormally elevated dALFF values in the angular gyrus found in PACG patients in this study may be twofold. On the one hand, there is a neural compensatory mechanism whereby reduced visual input may force the angular gyrus to enhance multimodal integration functions (e.g., dependence on auditory or tactile information), leading to increased fluctuations in local activity (101, 102). Similar mechanisms have been reported in diabetic optic neuropathy (DON), in which elevated prefrontal dALFF is associated with reallocation of attentional resources (103). On the other hand, chronic IOP elevation may lead to transsynaptic degenerative changes, and the angular gyrus, as a multimodal hub, may be abnormally excited due to input imbalance (e.g., visual- proprioceptive mismatch) (102, 104). Furthermore, PACG is not only an ocular disorder, but also involves neurodegenerative changes throughout the brain. Its patients have reduced functional connectivity of the default mode network, salience network, which may lead to abnormal functioning of the angular gyrus in memory and contextual integration (81, 101). elevated dALFF may predict early cognitive impairment risk, especially in semantic memory and situational recall tasks, and its correlation with PACG progression needs to be verified in conjunction with longitudinal studies (43, 105). Therefore, further studies are needed to clarify its clinical significance and specific differences with other cognitive disorders.

“Regulation of neuron projection development” and ‘membrane organization’ affect the pathological process of PACG from the dimensions of neuronal morphology and function establishment and membrane dynamic balance, respectively. The “Regulation of Neuron Projection Development” pathway is involved in the morphogenesis, orientation and functional establishment of neuronal axons and dendrites through the cascade of transcription factors (106), cytoskeletal and migratory regulation (107), and functional diversity establishment (108). This optic nerve axonal degeneration plays a major role in the pathophysiology of PACG. For example, axonal damage in retinal ganglion cells (RGCs) of PACG patients may be associated with SARM1-mediated NAD + depletion (109). Furthermore, aberrant phosphorylation of tau proteins leads to decreased microtubule stability, which likewise exacerbates axonal transport impairment (110). Inflammatory factors (e.g., IL-6) also inhibit axon regeneration-related gene expression through the MAPK pathway (111, 112). The “Membrane Organization” pathway encompasses membrane structure dynamics, lipid-protein interactions and signaling (113–115). Previous studies have suggested that trabecular meshwork extracellular matrix (ECM) remodeling (e.g., COL11A1 mutation) is dependent on the integrin signaling pathway and that structural abnormalities in membrane lipid rafts may interfere with integrin-ECM interactions, resulting in blocked atrial water circulation (116). This blockage of the atrial water circulation pathway is a key factor in the development of PACG. In addition, oxidative stress markers such as MDA and AOPP are elevated in the serum of PACG patients, suggesting that membrane lipid peroxidation may impair trabecular meshwork cell membrane function and exacerbate IOP fluctuations (117). Future studies need to further integrate transcriptional regulation, membrane signaling networks, and microenvironmental interactions to develop multi-target therapeutic strategies.

Specific expression analysis revealed that genes negatively correlated with PACG are greatly involved in most stages of thalamogenesis. The development of thalamus occurs in stages of neurogenesis, nucleus differentiation, axon guidance and establishment of thalamo-cortical connections. Thalamic neurons are formed during the embryonic period, with differences in gene expression in different nuclei determining their functional specificity (118); this is followed by a gradual development of axon guidance and connection establishment (119); and reciprocal thalamo-cortical connections continue to be perfected during the postnatal period (120). There are overlapping genes between the PACG and thalamic development, such as COL11A1, PLEKHA7, and CNTNAP5, and abnormalities in gene regulation affect the development of the thalamic nuclei (48, 121). Besides, the lateral thalamic geniculate body (LGN) is a relay station for retinal signaling to the cortex. Developmental axonal misorientation or abnormal synapse formation may result in decreased visual information processing efficiency and increased optic nerve sensitivity to elevated IOP (122, 123). This visual information processing disorder may influence the development of the thalamus after birth. Further exploration of brain-eye interaction mechanisms is needed in the future to provide new targets for early diagnosis and intervention of PACG.

PPI analysis revealed that both PLS + and PLS- genes associated with PACG could be constructed into a PPI network, in which the genes with the highest degree values were NOP58 and KRAS, respectively. NOP58 is a key protein involved in ribosome biosynthesis in the nucleolus accumbens, and forms a complex with NOP56 and NOP1 to regulate the processing and methylation of rRNA precursors (124). Obstruction of ribosome synthesis may exacerbate mitochondrial dysfunction, leading to reactive oxygen species (ROS) accumulation, which in turn damages the trabecular meshwork or iris stromal cells (111). KRAS, as a member of the RAS family, regulates cell proliferation, survival, and metabolism through pathways such as MAPK, PI3K/Akt, and others (125–127). Down-regulation of KRAS inhibits the Hippo pathway activity of the effector molecule YAP, and the Hippo pathway is involved in eye development and anterior chamber structure formation (128). Alternatively, KRAS silencing inhibits oxidative stress-induced iron death and may alleviate oxidative damage in trabecular meshwork cells, thereby maintaining the integrity of the atrial aqueous efflux pathway (126). Both NOP56 and KRAS may affect the synthesis of collagen eggs and matrix metalloproteinases, which both play key roles in iris stromal remodeling and atrial angle adhesion in PACG (48, 116). The synergistic effect of the two may provide new perspectives on the multi-mechanism pathogenesis of PACG, but further experimental validation is still needed.

5-HT4R is expressed in the ciliary body, iris, and choroid (129), and increases cAMP levels through activation of adenylate cyclase (AC), which in turn regulates downstream pathways such as PKA and Epac (130, 131). This mechanism may affect the atrial fluid secretion function of the ciliary body. In addition, 5-HT4R is involved in the regulation of ciliary and ophthalmic artery contraction. Obstruction of atrial aqueous outflow is a central pathologic feature of PACG, and alterations in vascular tone may indirectly affect the dynamic balance of intraocular pressure (IOP). On the other hand, 5-HT4R activation reduces astrocyte responses and inflammatory mediators such as IL-1β, and chronic inflammation is an important causative factor of optic nerve damage in glaucoma (130, 131). mGlu5R hyperactivation enhances NMDA receptor-mediated excitotoxicity, and glutamate accumulation is a key mechanism of RGC death in glaucoma (132, 133). In chronic IOP models, mGlu5R may increase intracellular Ca^2+^ overload through the Gαq-PLCβ-IP3 pathway, leading to neuronal damage (134). Taken together, 5-HT4R and mGlu5R may be involved in the pathological process of PACG through multiple pathways such as IOP regulation, neuroprotection, and inflammatory regulation, and future studies need to explore their interactions and feasibility as therapeutic targets in depth.

Several limitations of this study remain to be elucidated: (1) Due to the relatively restricted sample size, the current findings may be significantly affected by sampling error, and subsequent studies should confirm the robustness of the results by enlarging the sample size. (2) The limitations of the temporal resolution of the dALFF parameter need to be emphasized - the metric simulates dynamic temporal evolution through a sliding time window simulates dynamic time evolution, which is still essentially a quasi-static metric, and its calculation process is susceptible to systematic interference from rs-fMRI-related confounding variables. (3) The architectural flaws of adopting the AHBA database are noteworthy, as the dataset covers gene expression profiles of the left hemisphere brain regions only, leading to potential bias in neuroimaging studies based on hemispheric asymmetry, especially for dALFF and other brain regions with functional The interpretation of biomarkers characterized by lateralization of brain regions, such as dALFF, needs to be kept cautious.

## Conclusion

Using dALFF analysis, the present study revealed extensive dynamic functional remodeling in the occipital visual cortex of PACG patients by systematically evaluating their dynamic functional variability over multiple time windows, and suggested that both visual pathway damage and neurological compensation exist in this population. By integrating genome-wide transcriptomic profiling and whole-brain quantitative analysis of neurotransmitter receptor/transporter spatial distribution patterns, the present study elucidated the potential association between cortical layer-specific functional abnormalities and alterations in the neuromolecular microenvironment, and laid the foundation of molecular neuroscience for the development of innovative therapeutic strategies for PACG.

## Data Availability

The raw data supporting the conclusions of this article will be made available by the authors, without undue reservation.

## Footnotes

^1^SPM12, https://www.fil.ion.ucl.ac.uk/spm/.

^2^ https://string-db.org/

^3^ https://hbatlas.org/

^4^ https://neurovault.Org/collections/1206/
